# Antihormonal agents as a strategy to improve the effect of chemo-radiation in cervical cancer: *in vitro* and *in vivo* study

**DOI:** 10.1186/s12885-015-1016-4

**Published:** 2015-01-27

**Authors:** Mariana Segovia-Mendoza, Rafael Jurado, Roser Mir, Luis A Medina, Heriberto Prado-Garcia, Patricia Garcia-Lopez

**Affiliations:** 1 Instituto Nacional de Cancerología, Subdirección de Investigación Básica, México D.F., 14080 México; 2 Posgrado en Ciencias Biológicas, Universidad Nacional Autónoma de México, México D.F., 04510 México; 3 Instituto de Física, Universidad Nacional Autónoma de México, México D.F., 04510 México; 4 Unidad de Investigación Biomédica en Cáncer INCan-UNAM, Instituto Nacional de Cancerología, México D.F., 14080 México; 5 Instituto Nacional de Enfermedades Respiratorias, Departamento de Enfermedades Crónico-Degenerativas, México D.F, Mexico

**Keywords:** Cervical cancer, Mifepristone, ICI 182,780, Cisplatin, Radiosensitizing

## Abstract

**Background:**

Over the past few years, the concurrent use of cisplatin-based chemotherapy and radiation therapy has dramatically improved the local response and increased overall survival in early-stage cervical cancer. However, for the advanced stages of the disease this standard treatment has proved insufficient. We investigated the capacity of Mifepristone and ICI 182,780, which are anti-progestin and anti-estrogen drugs, respectively, to act as chemo-radiosensitizing agents in cervical cancer cells and cervix xenografts.

**Methods:**

The effect of chemo-radiation alone or combined with Mifepristone or ICI 182,780 was evaluated in HeLa cells and with tumor growth in cervix xenografts. After concomitant chemo-radiotherapy, the effect of each of these antihormonal agents on apoptosis (determined by Annexing V assay) and the cell cycle phases were determined by flow cytometry. The expression of angiogenic factor VEGF in tumor samples was determined using quantitative RT-PCR analysis of VEGF gene expression.

**Results:**

Compared to radiation alone or radiation/cisplatin therapy, there was significantly higher cytotoxicity and a greater antitumoral effect with the combined application of radiation/cisplatin and Mifepristone or ICI 182,780. Analyses of the apoptosis and cell cycle demonstrated changes only with ICI, not with Mifepristone, when was applied in combination with radiation/cisplatin. The analysis of VEGF mRNA expression levels in tumors at the end of the study demonstrated a significant inhibition, compared to radiation only or the radiation/cisplatin treatment, after concurrent chemo-radiotherapy and each one of the antihormonal drugs.

**Conclusion:**

Mifepristone and ICI 182,780 may be potentially promising chemo-radiosensitizing compounds to be used in combination with ionizing irradiation and cisplatin in the treatment of patients with advanced cervical cancer.

## Background

Cervical cancer is still a major cause of morbidity and mortality in women worldwide. In several low-income countries this disease, if not treated in time, is one of the most aggressive gynecological cancers. Although routine screening programs have been implemented since 1975, there continues to be an increased rate of new cases [1,2]. Human papillomavirus (HPV) has been proposed as an etiological factor in the pathogenesis of this cancer [3].

At present, cervical cancer is considered as a potentially curable disease in cases of early detection. Unfortunately the majority of cases are diagnosed in the metastatic or advanced stage, implying a worse prognosis that requires a systemic treatment. Currently, the treatment for cervical carcinoma is the combination of cisplatin-based chemotherapy and radiotherapy [4,5]. The introduction of chemo-radiation for early stage cervical carcinoma led to improvements in survival. Different studies have demonstrated that chemo-radiotherapy lead to a significant survival advantage of 10-15% at 5 years post-treatment compared with radiotherapy alone for patients in stage IB-IIB [6]. However, the prognosis for patients in stage IIIA-IVA is still unfavorable [6-8].

Apart from their limited effectiveness in the advanced stage of the disease, current chemotherapy and radiotherapy have serious drawbacks. The administration of cisplatin is associated with serious side effects, such as nephrotoxic and neurotoxic events [9], and the effectiveness of radiation therapy is limited by damage to the normal tissue. Several studies have sought to find drugs capable of potentiating the antiproliferative effect of chemo-radiotherapy in cervical cancer. In spite of the promising anti-tumoral results with the use of hydroxyurea, gemcitabine or taxanes as radiosensitizers, even greater side effects have been found with these drugs. Hence, ongoing efforts are necessary to further improve the outcome with locally advanced cervical cancer by maximizing the local response and patient survival.

One proposal of this work is to seek chemo- and radio- sensitizer drugs with less adverse effects. Some antihormonals, such as tamoxifen, raloxifene, medroxiprogesterone, mifepristone and ICI 182,780, have been used in the treatment of hormone-dependent cancers (e.g., breast, ovarian, prostate and endometrial). However, these compounds have been poorly studied as chemo-radiosensitizers in cervical cancer because this carcinoma is traditionally considered not to respond to antihormonal therapy [10]. Nevertheless, the mechanisms of action of these antihormonal agents described in the literature seem to hold promise in facilitating the response to chemo-radiation in cervical cancer cells. Such mechanisms include caspase activation, growth factor regulation [11], anti-apoptotic proteins inhibition [12,13] and apoptosis induction by p53 [14,15].

The possible role of mifepristone (MF) or ICI 182,780 (ICI) to enhance the cytotoxicity of cisplatin and the effectiveness of radiotherapy in cervical cancer treatment has not been sufficiently explored. MF has activity on progesterone and is a glucocorticoid receptor antagonist. It has been used a chemosensitizing drug to modulate the cytotoxic activity of doxorubicin [16], paclitaxel [17] and cisplatin on ovarian cancer cells [18]. We previously demonstrated that MF was able to enhance the cytotoxicity of cisplatin in cervical cancer cells *in-vitro* and *in-vivo* by increasing the intracellular and intratumoral concentration of cisplatin [19]. Furthermore, we recently reported a reduction in the rate of tumor growth when MF was added to the temozolamide-radiation scheme in glioblastoma xenografts [20]. Overall, this evidence suggests that MF could play an important role as chemo- and radiosensitizer.

In another study using cervical cancer cells [21], we showed that the combination of cisplatin with the antiestrogen ICI induced the arrest of the cell cycle at the G2/M phase. The failure of this control checkpoint may lead to genomic instability resulting in hypersensitivity to radiation.

The aim of the present study was to evaluate whether MF or ICI used simultaneously with cisplatin and radiotherapy could show a chemo-radiosensitizer effect, increasing the anti-proliferative effect in cervical cancer cells and in xenostransplants when treated with cisplatin and radiation. To correlate the mechanism of action of these antihormonals in the modulation of the effects of chemo-radiotherapy on tumor cells, an analysis of cell cycle and apoptosis at different times was made (the latter evaluated by Annexin V binding assay). Additionally, the growth of cervix xenotrasplants was correlated with a decrease in VEGF gene expression.

## Methods

### Drugs and reagents

Cisplatin, Mifepristone, Chloroform, Trypsin and Sodium Chloride were obtained from Sigma Chemical Co. (St. Louis, MO, USA). ICI 182,780 was obtained from Tocris Cookson, Inc. (Balwin, MO, USA). Dulbecco’s modified Eagle’s medium (DMEM), FCS (fetal calf serum), EDTA (Ethylenediaminetetracetic acid), Tris and SDS were obtained from Gibco, BRL (Grand Island, NY, USA). High-quality water employed to prepare solutions was obtained through of a Milli-Q Reagent Water System Continental Water Systems (El Paso, TX, USA). Taq DNA polymerase was purchased from Invitrogen (Carlsbad, CA, USA).

### Solutions

A stock solution (1 mg/mL) of cisplatin was prepared in saline solution. ICI and MF were reconstituted in absolute ethanol (stock solution). All standard solutions were stored at −20°C until use.

### Animals

Female athymic Balb-c nu/nu mice, between 6–8 weeks of age, were supplied by the Instituto Nacional de Nutrición (INCMNSZ), Mexico City, Mexico. All animals were kept in a pathogen-free environment and fed *ad lib*. The procedures for care and use of animals were approved by the Ethics Committee of the Instituto Nacional de Cancerología (INCan, Mexico City, Mexico) and all applicable institutional and governmental regulations concerning the ethical use of animals were followed.

### Cell cultures

The HeLa human cervical cancer cell line was obtained from ATCC (Rockville, Maryland, USA), and was routinely maintained as a monolayer in DMEM supplemented with 10% fetal bovine serum, and incubated at 37°C with high humidity in a 5% CO_2_ atmosphere. Cells were harvested with 0.025% trypsin and 1 mM EDTA.

### Chemo-radiotherapy on HeLa cells

Cells were seeded into 25 cm^2^ culture plates at a density of 5×10^5^ cells per well and pretreated for 3 days with 10 μ*M* MF or ICI. Control cells were exposed only to the vehicle (the final ethanol concentration never exceeded 1% v/v in treated or control samples). At the end of the exposure period, the culture medium was removed and fresh medium with 0.33 μ*M* of cisplatin plus MF or ICI was added. After 24 h the cells were irradiated at 0.75 Gy using a ^60^Co irradiator (Theraton, Phoenix, USA). Controls were handled in the same way as the irradiated cultures. Moreover, cells exposed only to the individual treatments (with or without radiation) served as a positive control. After exposure, cell survival was determined by the clonogenic assay.

### Clonogenic cell survival assay

Surviving cells were analyzed for cytotoxic effects of chemotherapy and/or irradiation treatments, according to the establish method of the clonogenic assay [22]. Briefly, following exposure to radiation, the cells were immediately rinsed, trypsinized, diluted, counted, and seeded in triplicate at different cell densities in 25 cm^2^ plates, and then allowed to grow at 37°C for 2 weeks. During this time colonies were formed from surviving cells, and these colonies were fixed in 10% formaldehyde and stained with a solution of crystal violet. Colonies of 50 cells or more were counted manually with a cell counter (Bantex, USA). The survival rate was expressed as the plating efficiency (PE), which is equal to the average number colonies counted divided by the total number of cells plated a calculation that normalized clonogenic survival. At least three independent experiments were performed for each assay.

### Cell cycle analysis

The cells (5 × 10^4^) were synchronized and plated in specific medium following the previously described protocol. At 24, 48 and 72 h post-irradiation, the cultured cells were harvested and washed twice with PBS, then fixed with 70% (v/v) ethanol and stored at 4°C overnight. Afterwards, ethanol was removed and the samples were washed with PBS. Cellular DNA staining was performed with Guava Cell Cycle Reagent for 30 min (Guava Technologies, Millipore, Hayward, CA, USA). The data were collected and analysed by a Guava EasyCyte Flow cytometer with use of GuavaSoft software (Millipore, Hayward, CA), as a minimum 1×10^4^ cells were acquired. At least three independent experiments were performed.

### Annexin V staining assay for apoptosis

Phosphatidylserine externalization was analyzed using the Guava Nexin Kit. Cells were plated and treated as before mentioned, the Annexin V binding assay was conducted at 24, 48 and 72 h post-irradiation. Cultured cells were harvested and washed twice with PBS, and then resuspended in Guava Nexin Reagent, containing binding buffer, Annexin V-phycoerytrin (PE) and 7-aminoactinomycin D (7-AAD), incubated for 20 min at room temperature according to Guava protocol instructions (Guava Technologies, Millipore, Hayward, CA, USA). Analysis was carried out by flow cytometry using Guava EasyCyte, acquiring 5×10^3^ cells. Annexin analysis was performed with the GuavaSoft software (Millipore, Hayward, CA). At least three independent experiments were performed for each assay.

### Tumor xenografts

Mice were subcutaneously (s.c) inoculated with 5×10^6^ HeLa cells in both hind limbs. The limbs were selected as the site for tumor growth to minimize irradiation to organs. Weekly measurements of tumors were made after inoculation. Two perpendicular diameters were measured by using a caliper, and tumor volume was determined by using the following relation: V = π/6 × (large diameter × [short diameter]^2^). Once tumors had reached approximately 150 mm^3^, the animals were pair-matched in treatment and control groups and the treatments were initiated. Each group consisted of 5–6 tumor-bearing mice.

### Irradiation procedure

Animals were anaesthetized with 1–3% isoflurane in 100% oxygen by using an animal anesthesia inhalation unit (Bickford, Wales Center, NY, USA), and irradiated with an orthovoltage X-ray unit (D3225, Gulmay Medical Ltd., UK) as described previously [23]. Animals received fractionated doses of 0.5 Gy per day for 20 days (Monday through Friday for four weeks). The dose and schedule were selected in accordance with the dose–response curve constructed in a previous pilot study. This curve showed a 10 Gy dose as the ED_50_ (dose of radiation to achieve 50% growth inhibition). The X-ray beam was centered on the tumor lobe by using one of the different lead collimators [23], depending on the tumor size at the moment of irradiation.

### Chemo-radiotherapy in tumor xenografts

Animals selected for this study were divided into eight groups (n = 5-6 each) including: A) radiation treatment alone (0.5 Gy/day for 20 days); B) cisplatin treatment alone (3 mg/kg/week for three cycles, i.p.); C) MF alone (2 mg/kg/day, s.c.) or ICI alone (100 mg/kg/day, s.c.); D) irradiation combined with cisplatin; E) irradiation combined with MF or ICI; F) cisplatin combined with MF or ICI; and G) three treatments combined (cisplatin/radiation/ICI or cisplatin/radiation/MF). ICI and MF were administered in three cycles during three weeks; each cycle consisted of three consecutive days (Monday through Wednesday). Control animals received only the vehicle and no irradiation. Mice were weighed and the tumor volume was calculated every five days as previously described. The experiment was conducted during 10 weeks, at the end of which time all animals were weighed and euthanized.

### VEGF expression analysis by quantitative reverse transcription-PCR (qRT-PCR)

The effect of MF or ICI on the expression of angiogenic factors during concomitant chemo-radiotherapy was examined using qRT-PCR. VEGF expression levels in tumor tissue from cervical carcinoma xenografs were evaluated at the end of the study. Briefly, whole tumors were lysed and the total RNA was isolated from each tumor with a method based on guanidine isothiocyanate/phenol/chloroform extraction using the TRIzol reagent (Invitrogen Life Technologies), and then quantified with UV spectroscopy. After quantification, 200 ng of the total RNA was used in presence of the TaqMan® RNA-to-CT™ 1-Step Kit (Applied Biosystems) to perform one-step RT-PCR TaqMan Gene Expression Assays of vascular endothelial growth factor A (VEGF-A) (Hs00900055_ml, Applied Biosystems) by using a FAM probe and Endogenous Control Human GAPDH (4310884E, Applied Biosystems) with VIC. Real-time quantification was performed on a Spectrum 48 thermocycler Instrument (ESCO, Micro Pte Ltd, Singapore).

PCR reactions were carried out in a total volume of 10 μl. The reaction conditions were as follows: pre-incubation at 60°C for 15 minutes and 94°C for 5 minutes, followed by 40 cycles (amplification) of 94°C for 15 s and 60°C for 60s. Fluorescence emission spectra were monitored and analyzed. PCR products were measured at the threshold cycle (Ct), at which time specific fluorescence became detectable. The Ct was used for kinetic analysis and was proportional to the initial number of target copies in the sample. Analysis of relative gene expression was based on the 2^-ΔΔCt^ method and was carried out with three independent samples.

### Statistical analysis

Values are reported as the mean ± SEM (standard error of the mean). Statistical analysis was performed by using one-way analysis of variance (ANOVA) to compare tumor volumes between groups, using SPSS Base 20.0 software (SPSS Inc, Chicago, IL, USA). Differences were statistically analyzed using multiple comparisons between groups. A log transformation was applied to data to better satisfy the assumptions underlying the analysis. The means and standard errors were computed from untransformed data, but analysis of statistical significance (p < 0.05) was based on transformed data. When necessary, comparison of the means was Bonferroni-adjusted.

## Results

### Growth inhibition in HeLa cells after chemo-radiotherapy

**Figure 1:**
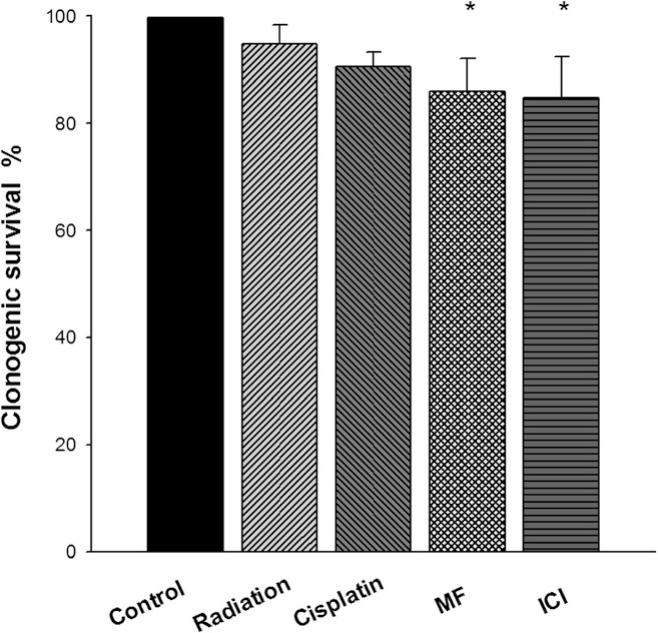
**Comparison of the clonogenic cell survival of HeLa cells after individual treatments.** The cells were treated with 0.75 Gy γ-irradiation, 0.33 μM cisplatin, MF and ICI (10 μM). Data are represented as the mean ± SD of three independent experiments. (*) Indicates a significant difference (p < 0.05) between the MF and IC groups vs control group.

The dose and the schedule of each drug used in the combination assays were selected in accordance with dose–response curves constructed in a previous pilot study. These curves showed that Cisplatin at 0.33 μM and 10 Gy of radiation did not show any inhibitory effect on the growth of HeLa cells when administered individually. However, it was observed a significant decrease (approximately 15%) with ICI or MF at 10 μM (Figure 1).

**Figure 2:**
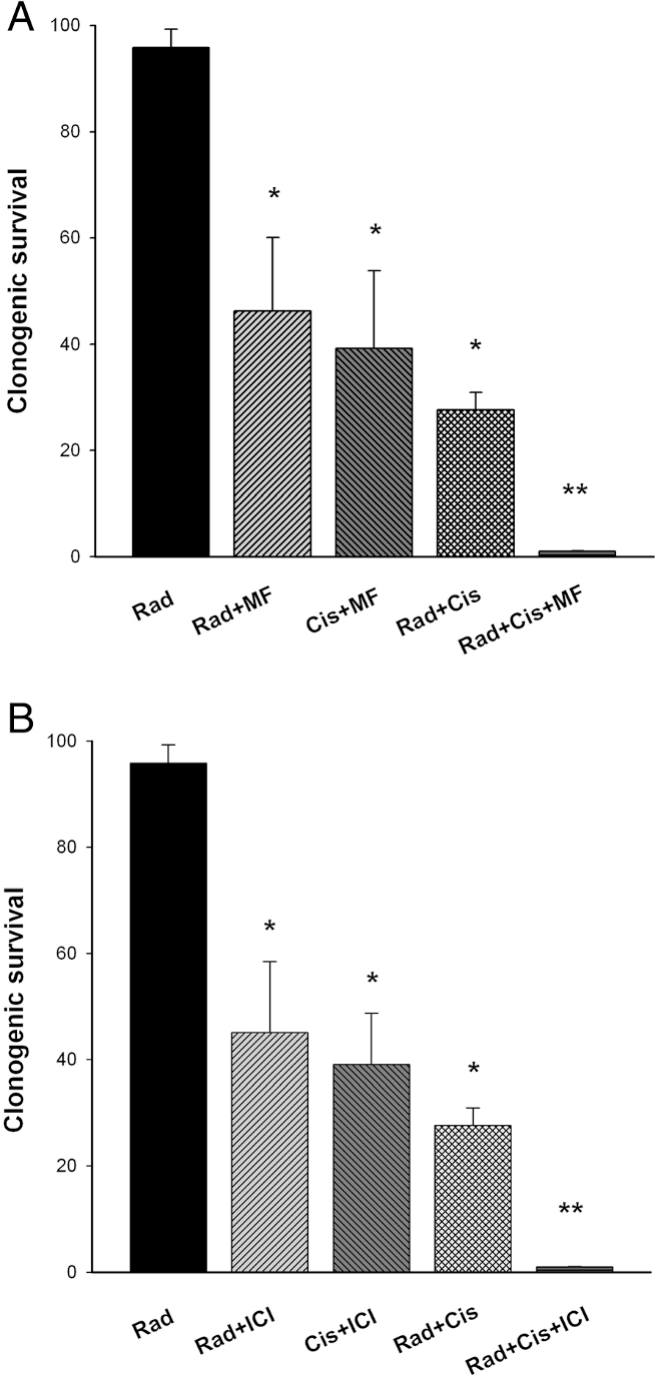
**Clonogenic cell survival on HeLa cells after combined treatments. A)** Combinations with Mifepristone. **B)** Combinations with ICI. All clonogenic assays were repeated in duplicate in at least three independent experiments. Values represent the mean ± SD.

Cell survival, evaluated by clonogenic assay, is shown for irradiation alone, irradiation with MF or ICI, cisplatin with MF or ICI, cisplatin with irradiation, and cisplatin with irradiation and MF or ICI (Figure 2). Irradiation alone had not effect on the cell proliferation. The antiproliferative effect was comparable when irradiation was applied in combination with cisplatin, or when either irradiation or cisplatin were combined with MF or ICI (about 30-50% of cell survival compared to the control) (Figure 2A, B). However, when the cells were exposed to a combination of irradiation, cisplatin *and* MF *or* ICI, the proliferation was almost completely inhibited. These experiments demonstrate that both agents can act as a chemo-radiosensitizer in cervical cancer cells.

### Cell cycle and apoptosis analysis

**Figure 3:**
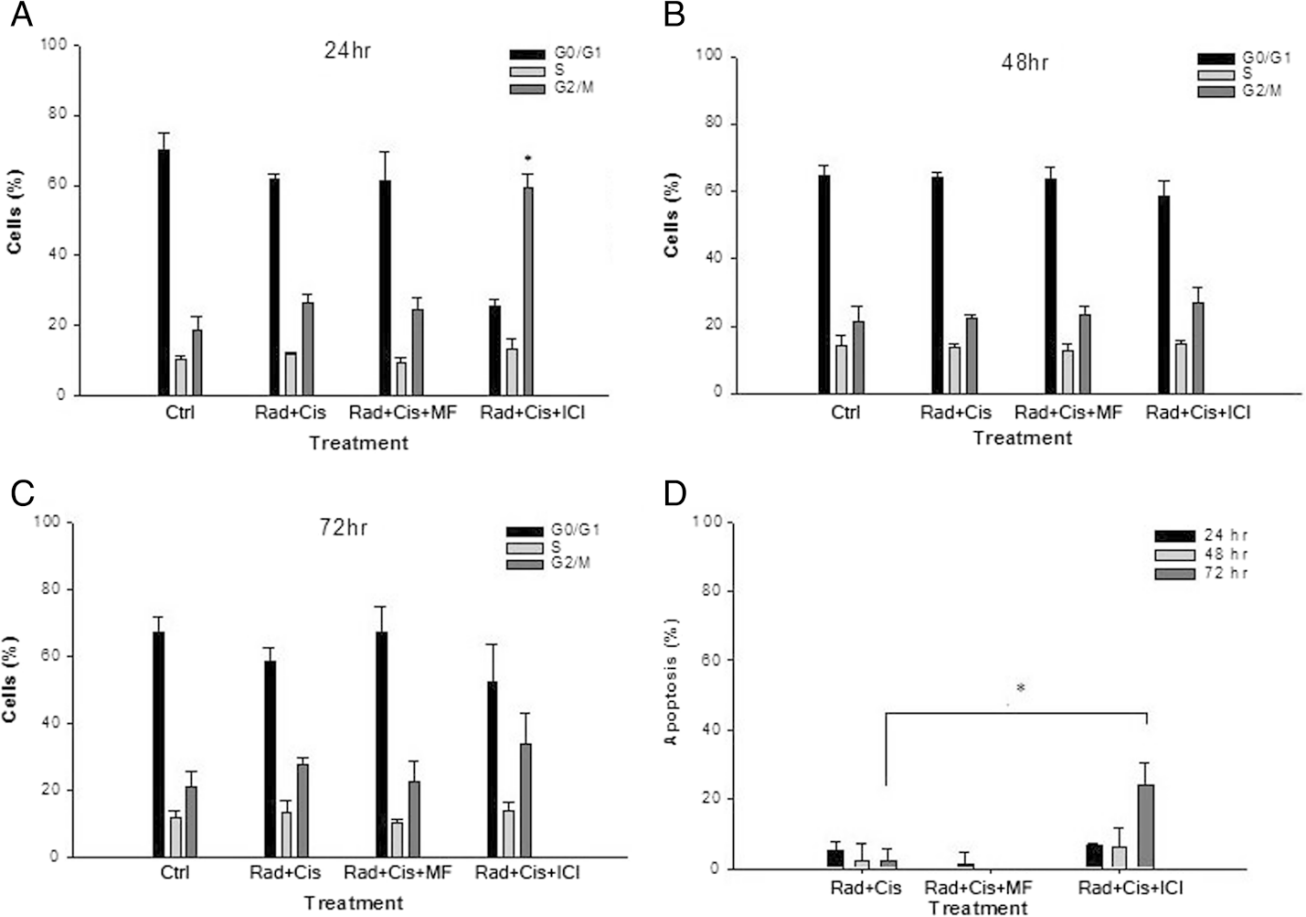
**Analysis of the cell cycle and apoptosis in HeLa cells after treatments.** Cell-cycle distribution was expressed as the percentage of surviving cells compared to the number of cells in the untreated control at 24 h **(A)**, 48 h **(B)** and 72 h **(C)**. Values represent the mean ± SD. *p < 0.05 (comparing MF or ICI combined with cisplatin/radiation treatment to the treatment with only cisplatin/radiation). Apoptosis was measured by surface AnV staining and flow cytometry, and it was considered positive for AnV+/PI- cells **(D)**. Values represent the mean ± SD *p < 0.05 (comparing MF or ICI combined with radiation/cisplatin to the treatment with only radiation/cisplatin).

To determine whether the effects of the combined treatments, using cisplatin and irradiation with MF or ICI on the proliferation of HeLa cells are mediated by inhibition of cell cycle progression, the cell cycle phases of treated cells were analyzed by flow cytometry at 24, 48 and 72 h. The results show that the G2/M phase was greater for the treatment with iradiation/cisplatin/ICI than for the treatment with only irradiation/cisplatin: 59.4% versus 26.4% at 24 h (Figure 3A; p < 0. 001), 27% versus 22% at 48 h (Figure 3B), and 34% versus 28% at 72 h (Figure 3C).

To assess whether growth inhibition by irradiation/cisplatin/ICI was mediated by apoptosis, HeLa cells were stained with Annexin V-PE and 7-ADD for flow cytometric analysis. Externalization of phosphatidylserine to the outer surface of the plasma membrane is a distinct phenomenon of early apoptosis. Analysis of Annexin V+/7-AAD- cells (early and late apoptosis) showed that the radiation/cisplatin/ICI treatment increased apoptosis at all times measured with respect to control cells. This change was particularly notable at 72 h, at which time the treatment with radiation/cisplatin/ICI increased apoptosis by 23.8%, whereas treatment with only radiation/cisplatin showed an increase of 2.36% increase compared to the control (Figure 3D; p = 0.020). Therefore, the addition of ICI arrested HeLa cells in G2/M phase at 24 and 48 h, and later (at 72 h) and induced apoptosis. These data suggest that G2/M arrested cells underwent apoptosis subsequently after treatment with radiation/cisplatin/ICI. On the other hand, cells exposed to radiation/cisplatin/MF did not show a significant change at any cell cycle phase or in regard to the induction of apoptosis (Figures 3A-D).

### Chemo-radiotherapy in tumor xenografts

**Figure 4:**
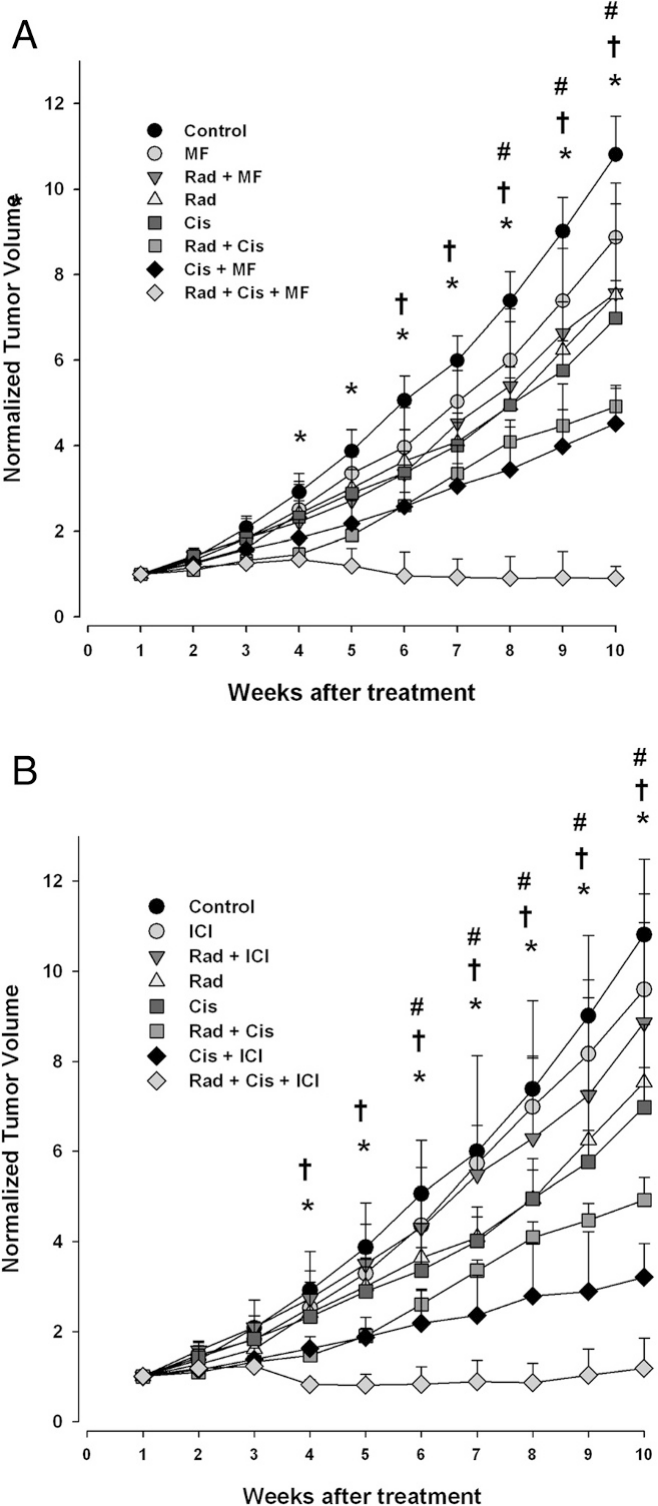
**Efficacy of antihormonal agents on HeLa cell tumors treated with cisplatin or radiotherapy.** HeLa cells were implanted s.c. in both hind limbs of nude mice. Treatment was initiated when the tumors reached 150 mm^3^. Mifepristone **(A)** or ICI **(B)** were administered in combination with radiation, cisplatin, and radiation/cisplatin. **B)** ICI was also administered in combination with radiation, cisplatin, and radiation/cisplatin. As controls, tumor growth was determined for mice treated only with the vehicle. Data are presented as the mean ± SEM of five to six animals. (*) indicates a significant difference (p < 0.01) between the triple combination groups vs control group; (#) and (†) represent a significant difference vs the dual treatments (p < 0.05) and individual treatments (p < 0.01) respectively.

Figure 4 shows tumor growth differences for the different treatment combinations: individual treatment (cisplatin, radiation *or* one of the antihormonals); dual treatment (radiation *or* cisplatin with one of the antihormonals); triple treatment (radiation *and* cisplatin with one of the antihormonals). Compared to the triple combination of radiation/cisplatin/MF, there was a statistical difference with the control group from the fourth week on (p < 0.01), with the dual treatments from the eighth week on (p < 0.05), and with the individual treatments from the sixth week on (Figure 4A; p < 0.01)*.* Whereas compared to the triple combination of radiation/cisplatin/ICI, there was a statistical difference with the control group from the fourth week on (p < 0.01), with the dual treatments from the sixth week on (p < 0.05), and with the individual treatments from the fourth week on (Figure 4B; p < 0.05).

**Figure 5:**
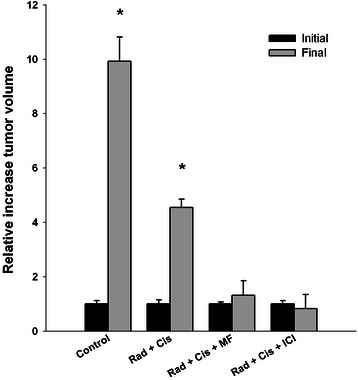
**Relative increase in tumor growth at the end of the study with respect to the initial volume.** *p < 0.05 comparing initial vs final.

After ten weeks, it was observed that in the radiation/cisplatin/MF and radiation/cisplatin/ICI groups, the tumor volume was not different from its initial volume (Figure 5). Contrarily, in the control group the tumor volume was 10-fold greater than the initial volume and in the radiation/cisplatin group it was 5-fold greater.

**Figure 6:**
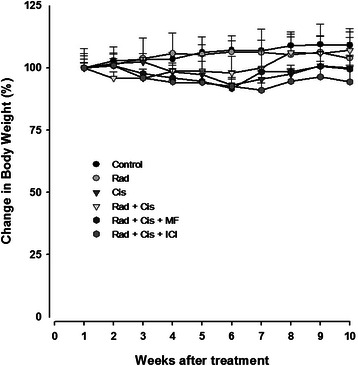
**Percentage of change in body weight for mice treated with cisplatin or radiotherapy, as well as for the combinations with each of the two antihormonal agents.** There was no significant difference between groups. Data are presented as the mean ± SEM of five to six animals.

Regarding the toxicity of treatments (Figure 6) no significant change in weight was observed, although there was a tendency to weight loss with the dual and triple treatment groups. In the latter cases, the weight of the animals was recovered by the end of the study.

### VEGF relative expression

**Figure 7:**
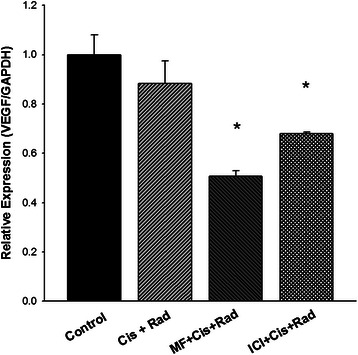
**Quantitative Real-Time PCR analysis of VEGF-A relative expression in HeLa xenografts after each treatment.** Analysis was done on whole lysated tumors removed at week 10. (*) Indicates a significant difference (p < 0.05) between the triple combination groups vs control group.

The effect of the triple treatments using cisplatin with one of the antihormonal compound on the VEGF mRNA levels was analysed in tumors at the end of the study. The triple combination of radiation/cisplatin/MF and radiation/cisplatin/ICI showed a statistically significant lower level of VEGF than that found in the control and radiation/cisplatin groups (Figure 7). Treatment with cisplatin and irradiation did not modify VEGF mRNA levels in tumors with respect to those from control group.

## Discussion

Currently, treatment for advanced cervical cancer involves the combination of cisplatin-based chemotherapy and radiotherapy. However, this treatment generates resistance and toxicity, either by the side effects caused by cisplatin (neurotoxicity, myelotoxicity, nephrotoxicity and ototoxicity) or the side effects caused by radiotherapy (damage to the normal tissue). Although this combination (cisplatin/radiation) has proved to be effective in the early stages of the disease, resulting in a patient survival rate of around 90%, its use for patients in advanced stages has led to a survival rate of only about 17% [7,8]. The poor response to treatment is mainly caused by chemo-radioresistant cells, which are capable of provoking uncontrolled local tumor growth.

Different efforts have been done to improve chemoradiotherapy for cervical cancer. However, there are few studies have attempted to identify potential agents as chemoradiosensitizer. Several compounds no-related to anti-hormonals agents have showed a potential radiosensitizer effect. Aspirin has showed radiosensitizing effect on human cervical cancer cells, its mechanism was mediated by Bcl-2, caspasas-3 pathway and p53 [24]. Plumbagin (naturally occurring naphtquinone) has been studied as radiosensitizer on HeLa, SiHa and C33A cells; results showed that this compound in combination with radiation augmented cell growth inhibition through of the apoptosis pathway [25]. Tillmans et al. [26] evaluated the radiosensitization of cervical cancer cell lines by retinoic acid demonstrating that the effect involves the HPV and p53 status. Furthermore, some drugs used in the chemotherapy as hydroxyurea, gemcitabine, taxanes have showed a radiosensitizing effect; however greater side effects can be found with these drugs.

In some types of hormone-dependent cancers, such as breast and ovarian, anti- hormonal agents have been used as chemo-sensitizer agents. However the application of these compounds as chemo-radio-sensitizers has been poorly studied in cervical cancer. Hence, the purpose of the present study was to investigate whether MF (an antiprogestin) or ICI (an antiestrogen) used simultaneously with cisplatin and radiotherapy could produce a synergism and increase the anti-proliferative effect in a cervical cancer cell line (HeLa) and in xenostransplants treated with cisplatin and radiation.

Prior to evaluating the effect of each agent, cisplatin and radiation doses were established for the combined assays. The concentration of drugs applied individually did not have a significant cytotoxic effect, thus a possible synergistic effect would only be found when combinations are applied. The individual dose of radiation and cisplatin used in the present study showed no more than 25% of cell death. The concentration of the antihormonals (10 μM), employed herein had no significant effect when these agents were applied individually. When this concentration, which is close to plasma concentrations achievable in humans, was used in previous studies, the anti- hormonal agent showed a chemo-sensitizer effect for cisplatin in ovarian [13] and cervical carcinoma cell lines [19,21], and for doxorubicin in hepatoma and leukemia cell lines [27]. This same concentration had a chemosensitizing effect with doxorubicin in breast-cancer cell lines [28] and a growth inhibition effect in ovarian cell lines [29].

Results of the clonogenic assay showed that MF has a chemo-radiosensitizer effect on the HeLa cell line. Cell survival decreased nearly 100% when MF was added to the standard treatment of cisplatin with radiation. It has been reported that MF inhibits proliferation of certain types of hormone-dependent cancers, such as breast cancer positive for the progesterone receptor [30,31], ovarian cancer [32,33], endometrium cancer [34], prostate cancer [35] and gastric cancer [36]. Cervical cancer does not respond to hormonal treatment. However, a study conducted in our laboratory demonstrated that when combined with MF, there was a significant increase in the cytotoxicity of cisplatin in two cervical cancer cell lines, HeLa and CaSki [19]. Tieszen CR et al. [37] reported that growth inhibition of cancer cells by antiprogestin MF is not dependent upon the expression of nuclear progesterone receptors. They showed that MF is capable of inhibiting the growth of in vitro cancer cells derived from the nervous system, breast, prostate, ovary and bone. Nearly all of these cancer cells lack the expression of classic nuclear progesterone receptors.

As a chemosensitizing agent, MF has been used to modulate the cytotoxic activity of doxorubicin, paclitaxel and cisplatin, mostly in hormone-dependent cancers such as breast cancer [16] and ovarian cancer [17,18]. However, there are few studies that demonstrate the role of MF as chemo-radiosensitizer agent. We previously reported that MF could improve the efficacy of chemo-radiotherapy in glioblastoma xenografts [20].

The addition of MF to the standard treatment of cisplatin with radiation did not induce significant changes in the cell cycle distribution. Previous studies have shown that MF induces G1-S blockage of the cell cycle through inhibition of cdk2 activity in human ovarian cancer cells [38]. A reduction in cdK2 activity has been associated with the inhibition of the transcription factor E2F1, which modulates S-phase progression [39]. The present study showed no changes in the G1 phase of the cell cycle when MF was added to chemo-radiotherapy treatment. Nevertheless, the dose of MF used in our study was lower than that used in previous reports. Furthermore, HeLa cell line shows low expression of hormonal receptors [21,40].

Many studies have reported the apoptotic effect of MF on different tumor types [41-47]. Recently it was reported that MF at low concentrations (<10 μM) could enhance the chemosensitivity of cancer cells to cisplatin, thus increasing the capability of this compound to induce apoptosis in HeLa cells. The greater effect of cisplatin on growth inhibition induced by MF was associated with the down-regulation of the HPV E6, survivin protein and the upregulation of the p53 protein [48]. Nevertheless, in the present study there were no significant changes in apoptosis when MF was added.

Whereas cell survival was reduced with the addition of MF to the cisplatin/radiation treatment (according to the clonogenic assay), no significant change was found in apoptosis or the cell cycle. The clonogenic assay involves a longer exposure (15 days) to treatment than that used in the analysis of apoptosis or the cell cycle (24 to 72 h). This could be one of the causes of the observed difference. On the other hand, when in the combined MF/cisplatin/radiation treatment involved a higher dose of cisplatin (3.3 μM), there was an increase in the percentage of cells in the G2/M phase at 72 h (data not shown). Therefore, exposure to MF for longer times or the use of higher concentrations of cisplatin could sensitize HeLa cell line by inducing G2/M arrest, the most radiosensitive cell cycle phase [49,50].

The results of the present study demonstrate that ICI also has a chemo-radiosensitizing effect on the HeLa cell line. The results show a decrease in survival with cells exposed to the combined treatment of ICI/cisplatin/radiation. The cytotoxic effect of the antiestrogen ICI in combination with certain antineoplastic agents has been clearly demonstrated in hormone-dependent breast cancer, reducing cellular proliferation and increasing cytotoxicity [51]. There is also evidence that the combination of cisplatin with ICI produces a synergistic effect, increasing cellular cytotoxicity in a negative estrogen receptor ovarian cancer line (A2780) [13]. A study conducted in our laboratory demonstrated that the combination of cisplatin with ICI produces a synergistic antiproliferative effect in cervical cancer cell lines. The effect of ICI on the cytotoxicity of cisplatin could be mediated, at least in part, by cell cycle arrest at the G2/M phase [21]. This correlates with the results obtained in the present study, where analysis of the cell cycle at 24 hours showed an increase in the percentage of cells arrested at G2/M when the ICI/cisplatin/radiation treatment was administered. Furthermore, there was an induction of apoptosis at 72 h. Therefore, the pathway of ICI to sensitize HeLa cell line to chemo-radiotherapy, is G2/M arrest preceding apoptosis, which may be a mechanism for its inhibitory effects on growth of the cancer cells. With the MF/cisplatin/radiation treatment, we hypothesize that the cisplatin dose should have been greater in order to arrest the cell cycle at the G2/M phase and induce apoptosis. The activity of MF appears to depend on the time of exposure to the treatment or the concentration of cisplatin used.

The response biological to progesterone is mediated by two isoforms of the progesterone receptor, PR-A and PR-B. In most cell lines such as MCF-7 (breast cancer cells), CV-1 (monkey kidney fibroblast), and HeLa (cervical carcinoma cells), PR-A functions as a transcriptional repressor, whereas PR-B functions as a transcriptional activator of progesterone-responsive genes [52]. Furthermore, it was reported that PR-A but not PR-B, in the presence of either progesterone or anti-progestin, inhibited ER-mediated transcriptional activity [53]. The same authors have reported that mifepristone was capable of functioning as an antagonist of ER only in the presence of PR-A in MCF-7 cells [52]. We previously reported that ERα and PR-AB gene levels in HeLa cells were relatively low compared to those observed in MCF-7 cells [21]; however, ICI was able to downregulated ER and PR genes, demonstrating a light ligand-receptor interaction. It is possible that MF could show similar results and its effect chemo-sensitization can be explained partially by this mechanism. Further studies will need to be performed to confirm this hypothesis.

In the present study we also evaluated whether there is an improvement in the response of xenotransplants of cervix *in vivo* with the addition of MF or ICI to the standard therapy of cisplatin and radiotherapy*.* We observed significant differences in tumor volume between the treatments. When the MF or ICI were combined with cisplatin, the effect was comparable to that of the standard treatment (cisplatin/radiation). However, there was a notable reduction in the tumor growth- rate when MF or ICI was added to the cisplatin/radiation scheme, suggesting that MF and ICI play an important role in the chemo-radiosensitization not only *in- vitro* but also *in- vivo.* When we evaluated the VEGF expression in the xenografts at the end of the study, we observed that both antihormonal agents decrease VEGF production. However, the effect was more evident with MF, suggesting that VEGF down-regulation is one of the mechanisms by this compound acts in combination with cisplatin/radiation.

Other mechanisms independent of ER/PR that could be involved in the chemoradiosensitizing effect of MF and ICI are the decreasing the insulin-like growth factor receptor (IGF-1), decreasing transforming-growth factor-β1 (TGF-β1) or loss c-fos expression.

On the other hand, it has reported that some tumour suppressor genes are involved in the regulation of angiogenesis. One of them, p53, has been shown to be associated with VEGF in non-small cell lung cancer [54]. The induction of VEGF gene expression by hypoxia in tumor cells involves both an increase in the rate of gene transcription mediated by HIF-1 (hypoxia-inducible factor), and an enhancement of the stability of VEGF mRNA. Transcription of VEGF mRNA is also induced by a variety of growth factors and cytokines, including PDGF (platelet-derived growth factor), EGF (Epidermal growth factor), TNFα (tumor necrosis factor), TGF-β1 (Tumoral growth factor), and IL-1β (interleukin) [55]. Also other genes as Bcl2 have been associated with neo-angiogenesis and a worse prognosis [56]. Moreover the loss of intracrine VEGF signaling leads to an increase in spontaneous apoptosis and chemosensitivity. These effects were mediated via upregulation of the proapoptotic mediators as caspase-3, and Bax [56].

Recent studies have showed that tumor cells express TLRs (Toll-like receptors), and this expression can facilitate the tumor development [57]. However, the relationship between TLR-8 and cervical cancer has been little studied. Recently, it has been reported the involvement of TLR-8 and its relationship with VEGF in cervical cancer [58]. The authors demonstrated increased expression of TLR-8 in HeLa cells and in cervical cancer tissue from patients; in this study also was evaluated the correlation between TLR-8 expression and two genes associated to the pathogenesis of cancer like Bcl-2 and VEGF. They found a positive correlation between TLR-8 and Bcl2 or VEGF expression both in cervical cancer tissues as well as HeLa cells. These results suggest that TLR-8 may be a therapeutic target in cervical cancer and its ligand can modulate the response of chemotherapy or radiotherapy. According to our results, it would be interesting to see in future studies, if the pathway TLR-8 is involved in the low expression of VEGF by the triple combinations of radiation/cisplatin/MF and radiation/cisplatin/ICI.

Finally, the lack of significant change in body weight of animals, suggest that both Mifepristone and ICI could be safely administered at these doses.

## Conclusions

The present study shows that the addition of Mifepristone or ICI improves chemo-radiotherapy treatment. It is possible that either of these antihormonal treatments sensitizes to HeLa cell line by inducing G2/M arrest, the most radiosensitive cell cycle phase, and by enhancing the capacity of cisplatin to induce apoptosis. Hence, the results strongly suggest that either antihormonal agent, when used in combination with cisplatin and radiation, could have potential as a chemo-radio-sensitizer for cervical cancer treatment.

## Abbreviations

HPV: Human papillomavirus
MF: Mifepristone
ICI: ICI 182,780
DMEM: Dulbecco ´s modified Eagle’s medium
EDTA: Ethylenediaminetetracetic acid
PE: Plating efficiency
s.c: Subcutaneously
VEGF: Vascular endothelial growth factor
RT-PCR: Reverse-transcriptase polymerase chain reaction
TC: Threshold cycle
cdk2: Cyclin-dependent kinase 2
